# More than Dysbiosis: Imbalance in Humoral and Neuronal Bidirectional Crosstalk Between Gut and Brain in Alzheimer’s Disease

**DOI:** 10.3390/ijms27010369

**Published:** 2025-12-29

**Authors:** Gauhar Tassibekova, Manzura Zholdassova, Nataliia Novosolova, Tarja Malm, Rashid Giniatullin, Almira Kustubayeva

**Affiliations:** 1 Department of Biophysics, Biomedicine and Neuroscience, Al-Farabi Kazakh National University, 050040 Almaty, Kazakhstan; tasibekovagauhar24@gmail.com (G.T.);; 2 Brain Institute, Al-Farabi Kazakh National University, 050040 Almaty, Kazakhstan; 3 A.I. Virtanen Institute for Molecular Sciences, University of Eastern Finland, FI-70211 Kuopio, Finland

**Keywords:** gut, gut–brain axis, microbiota, dysbiosis, metabolites, motility, neuroinflammation, AD

## Abstract

The intestinal microbiota, a diverse community of microorganisms residing in the human gut, recently attracted considerable attention as a contributing factor to various neurological disorders, including Alzheimer’s Disease (AD). Within the established framework of the gut–brain axis (GBA) concept, it is commonly suggested that dysbiosis, through microbial metabolites entering the brain, affect the cognitive functions in patients with AD. However, evidence for such a role of dysbiosis remains largely associative, and the complexity of the communication channels between the gut and the brain is not fully understood. Moreover, the new players of the GBA are emerging and the AD concept is constantly evolving. The objective of this narrative review is to synthesize the current evidence on the humoral, endocrine, immune, and neural communication mechanisms linking the gut and brain in AD and highlight newly discovered GBA messengers such as microRNAs, extracellular vesicles, T-cells, and the intestinal hormones, including emerging neuroprotective role for glucagon-like peptide-1 (GLP-1). Based on this knowledge, we aimed to develop a conceptual understanding of the GBA function in health and AD. We specify that, in AD, the GBA goes beyond a disrupted microbiome, but operates in conjunction with impaired intestinal secretion, motility, barrier permeability, and neuroinflammatory signaling. These factors are associated with the dysfunction of the hypothalamic–pituitary axis, altered somatic and autonomic neuronal gut regulation, and abnormal, due to memory problems, behavioral aspects of food intake. Identifying the individual profile of key molecular and cellular players contributing to an unbalanced GBA should optimize existing approaches or propose new approaches for the complex therapy of AD.

## 1. Introduction

The accumulated data indicates the importance of the interactions between the gut and brain within the concept of the gut–brain axis (GBA). These interactions are supposed to play a beneficial role in healthy states, but they can be converted into disease-promoting signaling in various pathologies. Among other common pathologies, in which the functional state of the GBA likely plays a significant role, is Alzheimer’s Disease (AD).

AD is a common neurodegenerative disorder, whose mechanisms are still debatable. It is generally accepted that the pathological hallmarks of AD are the presence of extracellular amyloid-beta (Aβ) plaques and intraneuronal neurofibrillary tau tangles [1]. Notably, while the amyloid burden still remains the leading hypothesis for AD, the failure of Aβ-targeting therapies [2,3] invites new views of the basic mechanisms of this common neurodegenerative disorder [4]. In this context, rethinking should also address the comorbidities and signals coming to the brain from other body systems, such as the gastrointestinal system.

Several recent systematic reviews considered different aspects of this complex topic. One comprehensive review summarized the data, showing bidirectional communication via the GBA in AD [5]. Another systematic review and meta-analysis provided data on the profile of the bacteria in AD patients [6]. The next comprehensive review discussed changes in the gut microbiota, and the role of immune cells [7]. The impact of gut-induced neuroinflammation in AD was further explored. Considerable interest was on gut-targeting treatment options in AD. While one systematic review presented data on the perspective of probiotics for AD treatment [8], another recent systematic review analyzed the results of other treatment options, such as fecal transplantation in patients and animal models of AD [9].

Thus, these reviews have focused on three main aspects of the GBA in AD: (i) dysbiosis, the profile of bacteria and harmful gut-derived metabolites; (ii) immune cells, inflammation, and neuroinflammation; and (iii) treatment options. Notably, there are much more complex processes in the gut, in which the secretion, digestion, motility, and neuronal mechanisms are closely interacting. Moreover, ongoing research is identifying new GBA signaling pathways, thus suggesting novel targets for the modulation of the gut-to-brain signaling. Finally, our understanding of the key processes underlying the onset and progression of AD is also evolving. A common shortcoming of many reviews in this field is the limited attention to the complex neuroglial mechanisms of AD. Overall, despite the abundance of attention to this topic, the general principles and concepts are currently lacking.

This narrative review focuses on identifying and integrating known and newly discovered multimodal bidirectional gut–brain signaling pathways into a coherent picture and highlighting newly discovered GBA messengers and the critical role of microglia in AD pathology. Based on these mechanisms and the general principles of the GBA, we discuss the potential promising options for AD treatment.

To this end, we conducted a literature search using the PubMed and Google Scholar databases, including research from its appearance until September 2025, and Research Gate was used as a supplementary tool. The search covered primarily research articles, including pre-clinical and clinical studies, and selected systemic and narrative reviews. The titles and abstracts were screened for the terms ‘gut–brain axis’, ‘Alzheimer’s disease’, ‘gut microbiota’, ‘microglia’, and ‘neurodegeneration’. The subtopics included terms ‘short-chain fatty acids (SCFAs)’, ‘bile acids (BAs)’, ‘extracellular vesicles (EVs)’, and ‘miRNA’. This approach was complemented by a subsequent review of papers citing articles found in the main search, to follow the development of the main stories and the confirmation of earlier findings and hypotheses. The main focus was on new signaling mechanisms and novel GBA messengers. Animal studies and, in particular, clinical studies were considered as the basis for interpretations and new hypotheses. The exclusion criteria included other neurodegenerative disorders such as Parkinson, Huntington diseases, and ALS. The selection of topics was hypothesis-driven, with an emphasis on emerging gut–brain signaling mechanisms relevant to microglial function in AD. We did not consider the role of astrocytes in AD, given that microglia, brain immune cells, appear to be the main target of gut metabolites. We also excluded articles describing the effect of prebiotics, probiotics, and postbiotics in AD, as these topics have been widely discussed in other sources. Articles not written in English and conference abstracts were excluded from our search as well.

## 2. Multimodal Bidirectional Gut–Brain Interactions in Healthy States

### 2.1. Humoral Communication Between Gut and Brain

**Figure 1:**
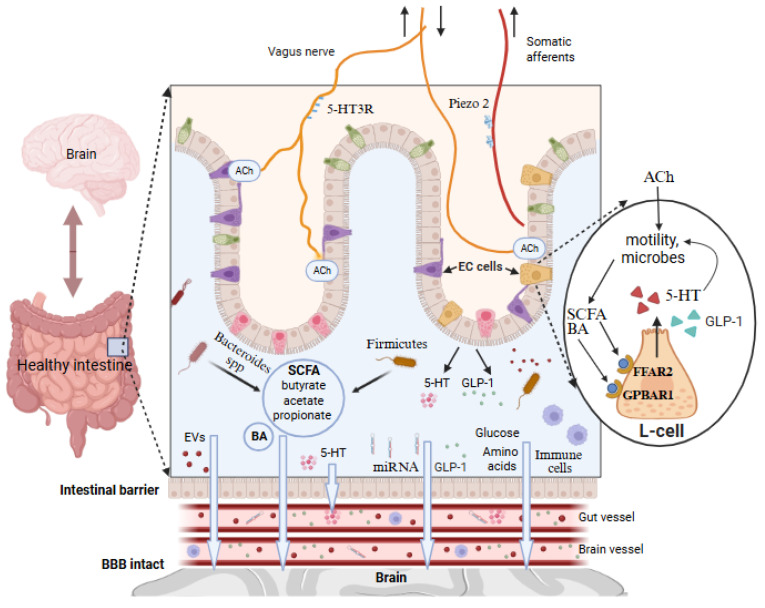
Gut–brain axis in healthy conditions. GBA is based on the bidirectional mechanisms including humoral and neuronal signaling. The intestinal microbiota represents an essential component of the GBA, producing metabolites such as SCFA, tryptophan (precursor of 5-HT), and bile acids (BAs), which connect the intestines to the brain, because, being lipophilic, some of these compounds could pass intestinal barriers and BBB. Glucose and amino acids as well as extracellular vesicles (EVs) can penetrate these barriers via specific transporters. Intestinal enteroendocrine cells (L-cells and EC-cells) respond to microbial and mechanical stimuli by release signaling molecules such as 5-HT, GLP-1 and other intestinal hormones (for details, see the main text). These messengers participate in the regulation of gut motility and send signals to the CNS through the vagus and somatic nerves, expressing 5-HT3, Piezo2, and a variety of other membrane receptors. Created in BioRender. Novosolova, N. (2025) https://BioRender.com/bj96bbm (accessed on 13 December 2025).

The current research permanently updates various types of communication channels for the two-way interactions between the gut and the central nervous system (CNS), known as the GBA. Although the gut and brain are physically separated, they are connected through various pathways, primarily via bloodstream, including the transport of intestine-derived molecules to the brain vasculature, where they may penetrate the brain parenchyma if they cross the blood–brain barrier (BBB; Figure 1). The chemical transmission of signals from the gut to the brain forms the most significant, and probably the most sensitive to pathologies, communication channel.

The human gut microbiota is a complex ecosystem of various microorganisms producing various biologically active metabolites. The essential class of these metabolites generated by the gut microbiota are short-chain fatty acids (SCFAs), such as acetic, propionic, and butyric acid [10]. In healthy conditions, a fraction of the SCFAs produced in the colon from carbohydrates enters the bloodstream (approximately 40% acetic acid, 10% propionic acid, and 5% butyric acid) to be distributed to peripheral tissues [11]. SCFAs are generated by a diverse gut microbiota, including members of the *phyla Bacteroidetes* and *Firmicutes* (Figure 1) [10,12]. SCFAs act locally, but, when taken to the bloodstream, they are involved in the regulation of such functions as metabolism, the modulation of the immune response, and appetite control [11], suggesting that SCFAs can reach the brain centers that control eating behavior.

In the context of the GBA, increasing attention was paid recently to the gut-produced primary and secondary BAs. After the BAs release from the liver, the intestinal microbiota can convert these primary BAs into secondary BAs, which may acquire new functions. Thus, being neuroactive lipophilic molecules, BAs can, along with SCFAs, be involved in the communication between the gut and the brain (Figure 1) [13,14].

**Figure 2:**
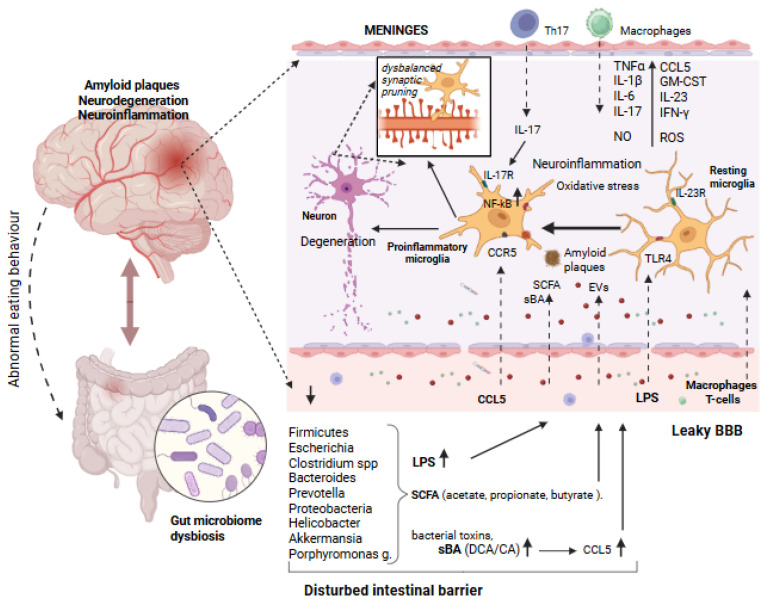
Disrupted gut–brain axis in Alzheimer’s disease. Intestinal microbiota composition in AD can lead to increased production of LPS, pathological variants of SCFA, neurotoxic secondary bile acids, and other microbial metabolites. When the intestinal barrier and BBB are disrupted, these harmful molecules pass more easily through the systemic circulation to the brain through the disrupted BBB. LPS entry into the CNS activates microglia through TLR4 and increases release of pro-inflammatory messengers (TNF-α, IL-1b, IL-6, IL-17, IFN-γ, ROS, and NOS), thus promoting neuroinflammation and oxidative stress. Activated microglia acquire phagocytotic properties to perform excessive pruning of dendritic spines, thus contributing to neurodegeneration. In contrast, microglia surrounding amyloid plaques lose their ability to remove waste products. Certain pathways, such as NF-kB, IL-17, and IL-23 activation, are also involved in pathological inflammation and neuron degeneration. The involvement of Th17 cells’ and macrophages’ migration into the brain becomes possible in AD through disrupted BBB or meninges and damaged glial limitans barrier. Activation of these multiple pathological mechanisms causes, along with neuroinflammation and oxidative stress, amyloid plaque deposition, and, finally, decline in synaptic transmission. Created in BioRender. Novosolova, N. (2025) https://BioRender.com/i4ojfu4 (accessed on 13 December 2025).

In addition to SCFAs and BAs, the GBA is complemented by signaling through immune cells, being conditioned in the gut wall, and then reaching the brain via the bloodstream. It has been proposed that some immune cells, such as T cells and pro-inflammatory T helper 17 (Th17) cells, can cause inflammation not only locally in the intestine, but also in the brain [15]. These authors demonstrated that, in inflammatory bowel disease, the pathogenic Th17 CD4+ T cells reached the brain, promoting neuroinflammation [15]. Notably, a diet rich in salt can increase the risk of cerebrovascular diseases and dementia via the expansion of Th17 cells in the small intestine, thus promoting interleukin-17 (IL-17) release [16]. While likely limited in healthy conditions, IL-17 can then enter the brain either through the disrupted BBB or via glial limitans, which are functionally linked to the meningeal lymphatic system, densely populated by various types of lymphocytes and other immune cells (Figure 2) [17,18].

Recently, new players in gut–brain communication have been proposed, such as microRNAs (miRNAs), generated in the crosstalk between intestinal cells and bacteria and released to the bloodstream, especially in the case of a compromised intestinal barrier [19,20]. This signaling pathway as a new candidate for GBA communications (Figure 1) looks attractive, consistent with the common signaling role of miRNAs in various functions, but further studies are required to confirm their involvement in GBA signaling.

Collectively, these findings illustrate that the concept of the GBA continues to evolve, with new molecular and cellular mediators expanding the list of humoral connections between the gut and brain in healthy conditions. However, for many members of this ever-expanding list of humoral messengers, the main limitation is the lack of direct evidence of their penetration across the BBB, as well as knowledge of the molecular and cellular targets of these messengers in brain cells.

### 2.2. Neural and Hormonal Connections Between the Gut and the Brain

The humoral communication within the GBA is complemented by neuronal mechanisms, involving autonomic and somatic nerves, as well as by the enteric nervous system (ENS). Within the complex gut innervation, the vagus nerve, containing both afferent and efferent fibers, plays a central role in the GBA, as a direct and fast communication channel between these organs.

One of the most interesting areas of research related to the role of the vagus nerve in intestinal innervation is new discoveries regarding the profile of membrane receptors on nerve endings that function as sensors of intestinal homeostasis. Thus, recently, Liu et al., suggested an original view, that, through the vagus, the gut reports to the brain a new ‘sense’, which allows the host to respond in real time to stimuli arising from the resident gut microorganisms [21]. This mechanism appears to involve microbial flagellin, which directly activates vagal nodose neurons to reduce feeding behavior [21]. Such an intriguing type of signaling can form a new ascending connection channel from the gut to the brain, whose role in health and disease deserves further studies.

The descending vagal activity is provided by the neurotransmitter acetylcholine (ACh), which controls motility, secretion, and other local gut functions (Figure 1). ACh can provide the beneficial so-called ‘cholinergic anti-inflammatory pathway’ (CAP) effect, due to the ability to dampen neuroinflammation in the brain [22], as well as peripheral inflammation in the intestine [23]. CAP has been extensively studied in brain pathologies, including AD, and accumulating evidence supports the notion that the cholinergic dysregulation and inflammatory responses play a key role in the pathogenesis of AD [24]. However, its importance for peripheral cholinergic systems as guardians of intestinal homeostasis requires further study.

In the gut, the local abundant neurotransmitter is serotonin (5-HT), produced by intestinal enterochromaffin cells (ECs) [25]. The release of 5-HT is largely dependent on gut motility, while, in turn, this transmitter coordinates secretion and gut motility, along with immune functions and the state of the intestinal barrier (Figure 1) [26,27]. This regulation of gut functions primarily involves serotonin type 3 receptors (5-HT3), expressed in vagal afferent fibers (Figure 1), sending neuronal spiking activity to the brainstem [28,29]. This means that peripheral serotonergic activity forms an important communication channel between the gut and the brain.

In the brain, 5-HT is well-known as a key signaling molecule, which modulates the mood and cognition, and shaping appetite, digestion, and emotional wellbeing [30,31]. This suggests that central serotonergic signaling may indirectly control gut function through the descending (from brain-to-gut) branch of the GBA. Thus, in both the brain and the gut, 5-HT is an important messenger that ensures the efficient functioning of the GBA under healthy conditions.

The vagal innervation of the gut is supplemented by less-explored somatic afferents, which, apart from sending pain signals, detect various intestinal functional states, in particular, motor activity, through recently discovered highly mechanosensitive Piezo channels [32] (Figure 1).

Thus, the complex neuronal network of the intestine, along with its relative independence from the CNS, is an emerging area of research that is likely to yield, in the near future, new discoveries of gut-to-brain interactions. Future studies are expected to expand our knowledge of the profile of membrane receptors in the vagus and somatic nerves, including mechanosensitive channels, which likely control most gut functions.

In addition to neuronal pathways, the GBA also includes the bidirectional hormonal control, when hormones produced by the gut modulate brain functions, while hormones produced by the hypothalamic–pituitary–adrenal (HPA) axis shape the functional state of the gastrointestinal system. Several studies suggest that the gut microbiota develops in parallel with the HPA axis, and that they are in constant interaction [33,34]. In some conditions, like irritable bowel syndrome, it has been shown that the common stress hormone cortisol directly affects immune cells and primary afferent nerve fibers in the gastrointestinal tract [35]. Notably, the stressful stimulation of the HPA can also increase the permeability of the intestinal wall and contribute to the development of dysbacteriosis [36,37].

As participants in the ascending loop of the GBA, intestinal enterochromaffin cells (EC) can secrete a variety of local hormones, such as gastrin, secretin, cholecystokinin, and peptide YY, which, apart from the control of gastrointestinal motility and food transit, can be taken into the circulation to approach other tissues and organs. The secretion of these intestinal hormones is controlled not only by the vagus and somatic nerve fibers, but also by locally produced BAs and SCFAs (Figure 1). Thus, the studies revealed that the G-protein-coupled BA receptor (GPBAR1) is highly expressed in the specialized L-type of EC cells, while the SCFA receptor free fatty acid receptor 2 (FFAR2) is present both in L-type and other EC cells (Figure 1) [38,39]. It means that the function of these hormone-secreted cells is under the control of these principal bacterial metabolites.

One recently proposed mediator of the interaction between the intestinal microbiota and the CNS is the glucagon-like peptide-1 (GLP-1). GLP-1, secreted from L-type cells in response to the nutrition type and microbiota activity (Figure 1), plays an important role in the regulation of glucose homeostasis and the energy balance, and exhibits pronounced anti-inflammatory properties [40,41]. A healthy diet that includes olive oil components, such as hydroxytyrosol, may promote the release of GLP-1 [42,43]. Through specific blood–brain barrier penetration mechanisms, GLP-1 can directly influence brain cells and promote neuroprotection [44]. This novel direct link could be supplemented by the GLP-1-analog–induced stimulation of pancreatic β-cells, mediating insulin secretion [45], which, via microglia, can promote anti-inflammatory effects in the brain [46]. However, the issue of whether the BBB is easily permeable by insulin under healthy conditions, or in disease, remains not entirely clear.

In summary, there are many protective humoral and neuronal mechanisms of the GBA that support healthy brain functions. The action of potentially harmful stimuli in the healthy conditions is minimized by structural factors, such as the intestinal barrier and BBB and the optimal coordination of secretion and digestion, as well as normal gut motility (evident as the lack of constipation), and supplemented by serotonergic cholinergic anti-inflammatory signaling. Table 1 summarizes the major gut-derived and host-regulated factors implicated in the GBA. Together, these multiple mechanisms form a kind of virtuous circle where the ascending and descending branches of the GBA are in optimal balance.

## 3. Unbalanced Gut–Brain Axis in AD

AD is a progressive neurodegenerative disorder and the most common cause of dementia, the pathophysiology of which still remains poorly understood despite numerous efforts in this area worldwide. Accordingly, not only are the diagnostic tools, in particular, AD biomarkers [65], continuously evolving, but treatment options as well [66]. In recent years, increasing attention in this field has been paid to the (dys)function of the GBA in AD [46,67]. The progress in the research in this area promises to identify new biomarkers and potentially innovative treatment options for AD. Conceptually, in the multi-step GBA, an imbalance at each step may be a potential contributing factor to the development of AD. Thus, such a contribution could be initiated by (i) dysbiosis, an abnormal profile of gut metabolites; (ii) a disrupted gut motility (and related poor chyme transit); (iii) a leaky intestinal barrier; (iv) the permeability of the BBB; and (v) changes in the functional states of brain glial cells, most notably, microglia, which are the most reactive responders to peripheral signals.

### 3.1. Dysbiosis in AD

One commonly discussed mechanism, proposed for an abnormal GBA in AD, is dysbiosis, leading to the production of toxic molecules and the activation of the immune system. Different species of Gram-negative bacteria are the primary candidates for dysbiosis, associated with the onset of AD [68,69,70]. Thus, *Helicobacter pylori* (*H. pylori*) infection was associated with neurodegenerative changes in cognitively normal men [71]. A long-term analysis of third National Health and Nutrition Examination Surveys (NHANES III) data has demonstrated an association between periodontal pathogens such as *Porphyromonas gingivalis*, *Prevotella melaninogenica*, and *Campylobacter rectus*, and an increased risk of incidence of AD [72]. Other studies showed that patients with AD have an increased proportion of *Bacteroidetes* type bacteria, while the content of the beneficial *Firmicutes* and *Bifidobacterium* types is significantly reduced [73]. The studies of patients with MCI and AD showed an increase in the *Gammaproteobacteria*, as well as *Enterobacteriales* and the *Enterobacteriaceae* family [74]. The meta-genomic sequencing in the preclinical stage AD demonstrated uprising levels of *Dorea formicigenerans*, Oscillibacter sp., *Faecalibacterium prausnitzii*, *Coprococcus catus*, and *Anaerostipes hadrus* [68] (Figure 1).

There are some conflicting data regarding the composition of the gut microbiota in AD [75,76,77,78]. For example, one study found that the content of *Bacteroidetes* was reduced in AD patients, while *Ruminococcaceae*, *Enterococcaceae* families, and *Lactobacillus* were increased [67]. These discrepancies are likely due to the differences in the study methodology, population characteristics, and disease stages, as well as environmental factors and diet of the participants.

An intriguing new aspect of the role of bacteria in the development of AD is based on recent research suggesting that, in addition to the local harmful effects on the gastrointestinal tract, pathogenic Gram-negative bacteria have the potential to penetrate the brain. This research suggested several proposed routes for how bacteria can enter brain. First, they could overcome the intestinal barrier and then destroy the intercellular junctions of endothelial cells in brain vessels, which allows them to cross the BBB through the paracellular pathway [57]. In particular, *E. coli* can bind to the receptors of endothelial cells to destroy the tight junctions between these cells, thus compromising the BBB [79]. Second, Gram-negative bacteria can cross the BBB by transcytosis, through interactions of bacterial proteins with the outer membrane of endothelial cells [58,80]. Finally, Gram-negative bacteria can enter the CNS along the cranial trigeminal and olfactory nerves [81,82]. Even though it remains not fully clear how common this phenomenon is, there are reports that the brain of patients with AD contains 5–10 times more bacteria than a healthy brain [83]. Although such bacterial enrichment of the brain could be an additional factor potentially contributing to the development of AD, evidence for such mechanisms remains weak, as it is mainly derived from in vitro models of the human BBB.

More strong evidence for the brain delivery is provided for bacterial EVs [84,85,86,87], since, apart from in vitro studies, this possibility was explored in animal models [87]. However, further studies demonstrated the presence of lipopolysaccharide (LPS)-containing EVs in the plasma of AD patients [88]. These authors also showed that such bacterial EVs can activate the excessive pruning of synapses via microglia activation [88], which could be a factor contributing to neurodegeneration (Figure 2).

Thus, although these data indicate a significant role of gut microbiota in the progression of AD, there is a variability in the observations obtained from different sources. The evidence for the link between dysbiosis and AD development remains mainly associative and the data on the movement of bacteria across the BBB in vivo are highly debatable, as they are based mainly on the evidence from in vitro studies. Together, this highlights the need for further systematic studies to more accurately establish the role of the abnormal microbiota and dysbiosis in the pathogenesis of AD, including the careful exploration of the bacterial and EV delivery from the gut to the brain.

### 3.2. Gut-Derived LPS as a Trigger of Neuroinflammation in AD

Out of other toxic compounds delivered by bacteria, the most attention is traditionally paid to LPS (Figure 2), produced by Gram-negative bacteria [89]. The important observation is that the plasma level of LPS is significantly elevated in patients with AD [59,89], consistent with the pro-inflammatory signaling caused by LPS in AD and amyotrophic lateral sclerosis (ALS) pathology [90]. These data are consistent with the current understanding of the contribution of the gut microbiota to neuroinflammation: LPS from Gram-negative bacteria is thought to enhance oxidative stress and pro-inflammatory responses, which may contribute to the progression of neurodegenerative diseases [91]. In the brain, the primary target for LPS is microglia (Figure 2), which normally quickly respond to changes in the brain environment to clean tissue from the waste, including accumulating Aβ [92]. LPS can contribute to the development of AD pathology by stimulating the phagocytic state of microglia via Toll-like receptor 4 (TLR4) and activated nuclear factor-κB (NF-kB) (Figure 2) [93,94,95], which leads to the secretion of multiple pro-inflammatory cytokines and the promotion of the oxidative stress (Figure 2). Apart from TLR4, LPS increases the expression of the receptor for Advanced Glycation End-products (RAGE), which is critically involved in the pathology of AD, including Aβ deposition, and cognitive impairment [96]. LPS activates the microglia via TLR4, switching them to a pro-inflammatory phenotype via the NF-κB pathway, causing neuroinflammation and cognitive impairment. The blockade of TLR4 with the VIPER peptide prevents these effects (Figure 2) [97]. Notably, the long-held “binary” view of microglial functional states (pro-inflammatory M1 and anti-inflammatory M2) is now being updated, suggesting multiple functional subtypes of these glial cells, associated with a given brain disease [98,99]. The latter suggests a promising new direction for further research concerning LPS targets in the brain.

Even though it is generally accepted as a highly pathogenic and pro-inflammatory compound, it is important to note that multiple species of Gram-negative bacteria produce different types of LPS, with distinct abilities to trigger inflammation. Interestingly, the fecal LPS from a healthy human microbiome, primarily from *Bacteroidales*, weakly activates TLR4 and can suppress cytokine responses induced by highly pro-inflammatory LPS, such as from *E. coli* [60]. This currently appears to be a controversial issue, as other studies report that *Bacteroides fragilis* can also produce a pro-inflammatory form of LPS [61].

Thus, the obtained data indicates several complementary molecular mechanisms for the action of LPS in AD, which, together, contribute to the development of this pathology. The potentially beneficial forms of LPS mentioned above are of considerable translational interest, but this unexpected inhibitory mechanism requires further study, particularly its association with the specific bacterial species.

### 3.3. Abnormal Profile and Complex Function of Gut Metabolites in AD

In a recent study, six types of SCFAs were found to be reduced in patients with MCI and AD, with valeric acid showing a particularly notable reduction in AD patients, while five other SCFAs, such as formic, acetic, propanoic, 2-methylbutyric, and isovaleric acids, were significantly different between the MCI and AD groups [100]. Likewise, in AD patients, fecal SCFAs propionate, isovalerate, and propionate-producing bacteria are inversely associated with amyloid status [101]. In the study employing a mouse model of AD, it has been established that SCFAs butyrate reduces Aβ levels and weakens cognitive dysfunction caused by AD [47], thus providing support for the beneficial role of SCFAs in AD. A Kazakhstan-based project ‘*Study of Gut Microbiota Alterations in Alzheimer’s Dementia Patients from Kazakhstan*’ revealed that, compared to age-matched healthy controls, patients with AD have significant alterations in microbiota, such as a decrease in SCFA-producing bacteria *Bifidobacterium and Roseburia* [102].

While the majority of data obtained from animal models and human studies suggest a beneficial effect of SCFAs in AD, it is important to note that certain studies have shown that some SCFAs may have a negative effect. Thus, Vinolo et al. found that the SCFAs acetate, propionate, and butyrate increased the release of the cytokine CINC-2αβ (cytokine-induced neutrophil chemoattractant-2αβ), promoting the migration of neutrophils to the site of inflammation, thereby aggravating the inflammatory response [103].

In summary, the available data, including studies in AD patients, indicate that SCFAs play a predominantly neuroprotective role in the pathogenesis of this disorder. Because much of the data regarding SCFAs has been obtained from animal studies and small groups of patients, further research and confirmation in larger patient cohorts is needed.

Studies show that patients with AD have changes in the profile of primary and secondary BAs. Thus, a decrease in the level of the primary BAs, cholic acid (CA), and an increase in the level of the bacteria-generated secondary BAs, deoxycholic acid (DCA), and its conjugates with glycine and taurine have been shown [48]. Notably, some studies showed that DCA and lithocholic acid (LCA), can cross the BBB and affect brain functions in AD (Figure 2) [48,49,50,104]. An increase in the DCA/CA ratio correlates with cognitive decline, suggesting a possible role of the gut microbiota in AD progression [49]. These data, taken together, suggest that BAs could be significant contributors to neurodegenerative diseases, such as AD [49,50,62].

One of the key questions with a significant translational impact is the origin of secondary BAs, and their link to certain gut bacterial taxa. In this regard, it has been shown that the proliferation of pathogenic *Clostridium* species, such as *C.difficile*, increases the level of DCA, which, in turn, elevates the serum level of C-C motif ligand 5 (CCL5) and induces CCL5 receptor 5 (CCR5) overexpression in sensory neurons [105]. Notably, the high expression of CCR5 in pro-inflammatory microglia was found in AD patients [106], and it has been proposed that CCR5 is one of the major hub genes in AD [107]. Based on these data and other studies showing a link between *C.difficile* and AD [108], we can speculate that such CCL5/CCR5 signaling could be involved also in the exaggeration of neuroinflammation in AD patients, having an ingrowth of *C.difficile* (Figure 2).

Thus, AD-associated changes in the composition of microbiomes and the progression of dysbiosis lead to an abnormal profile of BAs, increasing the levels of toxic secondary BAs, such as DCA and LCA, which can contribute to neuroinflammation and related damage to neurons (Figure 2). Taken together, it appears that the role of BAs in AD is more complex than that of SCFAs, as some BAs can have neurotoxic effects, requiring more careful consideration of the role of bacterial taxa that converts primary BAs to secondary ones.

These and other pathogenic contributors to the GBA are shown in Table 1.

### 3.4. Distorted Neuronal and Hormonal Control of the Gut in AD

The multiple neuronal pathways appear to be dysfunctional in AD, leading to a disruption in gut motility and gastrointestinal transit, as shown in a large cohort of AD patients, and additionally confirmed in the 5XFAD AD model mice [55]. Slow chyme transit, due to lowered gut motility, through the gut in AD, leads to constipation, which is a common symptom in this disorder [56]. Constipation in AD may also be due to the altered intestinal profiles of SCFAs and BAs, which interact with the neuronal control of intestinal motility (Figure 1).

The dysfunction of the ascending neuronal mechanisms may lead to a disruption in the flow of information from the digestive system to the brainstem and other brain areas that regulate food intake [109]. We speculate that irregular feeding behavior in AD, in return, results in abnormal motor/secretory activity in the gut, a deficiency in key nutrients, and the further progression of dysbiosis.

The precise mechanisms by which the intestine utilizes complex neuronal networks to coordinate vital gut functions, interacting with the brain, are not fully understood and remain an area of active research. In this context, a recent discovery demonstrated that the hippocampus contains specific centers that collect information transmitted by the vagus nerve and somatic afferents from the gut to control eating behavior [110]. Since the hippocampus is the brain region most prone to AD-related neurodegeneration, we can speculate that such pathological processes in the brain can affect the episodic memory associated with food intake.

Common symptoms of AD include weight loss and, as mentioned above, abnormal food-seeking behavior, functions also controlled by the hypothalamus. This brain region, crucial for the control of the metabolism, appeared to also be involved in the pathogenesis of AD [111]. Thus, Robison et al. (2023) found, in the 3xTg-AD mouse model, an increased expression in the hypothalamus of pro-inflammatory cytokines, suggesting the development of the local neuroinflammation [112]. Consistent with this, circadian rhythms, known to be dependent on hypothalamus activity, were already disrupted in a pre-symptomatic cohort of AD patients, along with other biomarkers of this pathology [113].

**Figure 3:**
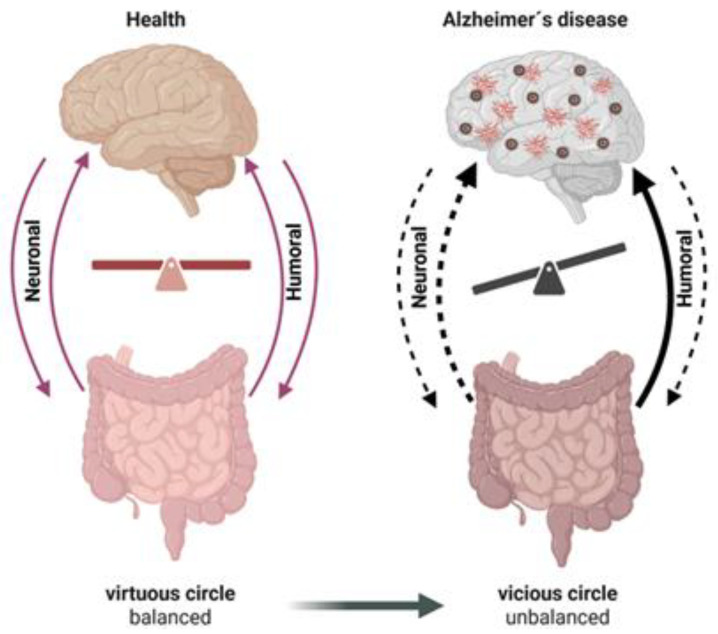
Proposed model of GBA representing transformation of the healthy balanced *virtuous circle* into AD-related harmful unbalanced *vicious circle*. In this model of bidirectional organ crosstalk, “neural” control involves direct bidirectional transmission of spiking activity through autonomic/somatic nerves and the cognitive control of eating behavior, which is often impaired in AD. ’Humoral’ interactions represent delivery of gut-produced metabolites and other messengers to the brain and neuroendocrine signaling. According to this concept, AD treatment options should strengthen weakened neural mechanisms and reduce harmful humoral connections transmitted to the brain from the gut. Created in BioRender. Novosolova, N. (2025) https://BioRender.com/hcns9am (accessed on 3 December 2025).

Recently, Kim et al. (2025) demonstrated the new potential channel of communication within the GBA, which relied on the detection of D-glucose in the gut by somatic (but not visceral) nerves [114]. This ascending signaling results in the modulation of the activity of corticotropin-releasing factor (CRF)-expressing neurons in the hypothalamic paraventricular nucleus. Notably, this paraventricular nucleus of the hypothalamus is undergoing degeneration in AD pathology [115,116,117], which may follow in deviant food behavior in this pathology, affecting multiple functions of the gastrointestinal system, ultimately further unbalancing the GBA (Figure 3). Notably, the dysfunction of the HPA and elevated cortisol can further promote hippocampal damage in AD [63], providing insight on how the interaction between these brain centers may contribute to the development of this disorder.

Overall, recent discoveries reveal a multitude of neural and humoral mechanisms linking the gut and the brain that acquire new deleterious functions in AD, forming a *harmful vicious circle*, which supports the pathological process.

## 4. Perspectives for AD Therapy Through the Gut–Brain Axis

Given the multi-step nature of bidirectional communication within the GBA, there are many potential targets for correcting defective mechanisms in AD. The proposed concept of a *harmful vicious circle* specific to AD suggests that the main strategy is to return it to a normal state of a *virtuous circle* (Figure 3). The potential molecular and cellular targets are illustrated in Table 1, Figure 1 and Figure 2. Within this strategy, the central point is to find a match between the GBA approach and AD-specific drugs, such as monoclonal antibodies against amyloid plaques, a reduction in neuroinflammation, and the promotion of cholinergic transmission, or other upcoming therapies.

### 4.1. Optimizing Microbiota in AD: Focus on Fecal Transplantation

Optimizing the microbiota to improve ascending gut-to-brain mechanisms in AD could be potentially achieved via diet, more specifically, with prebiotics, probiotics, and postbiotics, or by the fecal transplantation of beneficial bacteria. The former has been addressed elsewhere, and, in our review, we focused on fecal microbiota transplantation, which is an attractive but still rather controversial area of research.

Thus, it has been shown that the treatment of AD mouse with fecal microbiota from a person with a low risk of AD, due to the protective APOEe2 allele, improved memory but promoted neuroinflammation, probably due to the heterogeneous strain of bacteria in the host and donor microbiota [118]. In the other study, Jiang et al., before transplantation, used a prior depletion of endogenous strains by antibiotics and noted that the positive effect is temporal and reduces with time [119]. Similar results were observed by Grabrucker et al., who found that the microbiota from AD patients disrupted cognition and hippocampal neurogenesis into microbiota-depleted rats [120]. These studies mixing the native recipient with donor bacteria strains revealed several unexpected pitfalls in this procedure. Nevertheless, Sakurai et al., in a randomized placebo-controlled trial, showed beneficial effects of the *Lactiplantibacillus plantarum* OLL2712 strain in patients with memory problems [121], demonstrating that this procedure remains promising.

In this context of controversial research, an interesting alternative for the correction of the microbiome is the use of synthetic bacteria. Thus, a recent Science 2025 article reported the successful generation of *Escherichia coli* with a 4 Mb synthetic genome opens a potential perspective for enriching the microbiome by bacteria with designed properties [122]. Despite the originality of this approach and its potential coupling in future with AI technologies, its intervention in translational medicine is still far from practical implementation.

In summary, there are various pitfalls in fecal microbiota transplantation aimed at improving the GBA in AD, most notably a temporal beneficial effect of this procedure. As mentioned above, one of the complications is that the pathological strains of bacteria may be present in the recipient stool even after transplantation. It means that fecal microbiota transplantation requires the consideration of bacterial heterogeneity in both the recipient and donor, as well as the different roles of strains in different parts of the gastrointestinal tract.

### 4.2. Reinforce Positive and Diminishing Toxic Humoral Links

SCFAs produced by anti-inflammatory gut microbiota regulate immune responses by enhancing the differentiation of regulatory T cells and limiting the production of pro-inflammatory cytokines [123]. Chandra et al. proposed a propionate-based therapeutic strategy to attenuate Th17 signaling, leading to reduced amyloid deposits [124]. These exciting results are to be confirmed in clinical trials, before the application to AD patients.

BAs, particularly secondary BAs, play a predominantly pro-inflammatory role. This suggests that, unlike SCFAs, the therapeutic approach to these intestinal metabolites should primarily focus on reducing their pathogenic role in AD. While the profile of primary BAs is dependent on liver function, the level of secondary BAs is determined by the activity of gut microbiota, consistent with the data that brain BAs have a peripheral origin [125]. Consistent with the former, it is established that diet-induced alterations to liver function can affect brain Aβ homeostasis [126]. Thus, in AD, the treatment options may require optimizing the peripheral BAs metabolism both via diet and by normalizing microbiota.

Surprisingly, recent data suggest that some BAs could provide a positive effect in AD pathology. For instance, *Tauroursodeoxycholic acid* appeared to play a beneficial role in the prevention and possibly in the treatment of AD [127]. Furthermore, the BAs action via the Takeda G protein-coupled receptor 5 (TGR5) and the farnesoid X receptor (FXR) is of significant interest, as studies have demonstrated the anti-inflammatory effect of TGR5 activation, leading to the suppression of macrophage function and cytokine production, while FXR activation reduced intestinal inflammation and epithelial permeability. These data support the use of specific TGR5 and FXR modulators as potential therapeutic strategies for AD [128,129].

In summary, even though SCFAs and some BAs have emerged as promising therapeutic tools in AD, their therapeutic administration, due to their involvement in multiple aspects of AD pathology, requires further clinical research, considering the microbiota profile in the individual patient.

### 4.3. Testing GLP-1 as a Promising Treatment Option in AD

The ability of GLP-1 to penetrate the BBB and exert a neuroprotective effect by reducing neuroinflammation, modulating amyloidogenesis, and reducing oxidative stress [51] suggests that this gut-produced hormone can be a potential treatment option for AD.

Thus, the dual GLP-1/Gastric inhibitory polypeptide (GIP) receptor agonists such as DA-JC4, DA5-CH, DA-JC1, and DA-CH3 have shown the ability to reduce inflammation, and reversed memory impairments and enhanced synaptic plasticity in the hippocampus of APP/PS1 mice. In 3Tg AD mice treated with DA-JC4, improvements were observed in object recognition, spatial memory, and hippocampal pathology, including reductions in Aβ and tau accumulation.

Apart from animal models, clinical studies suggested that GLP-1 receptor agonists may help to slow cognitive decline in AD patients. For instance, phase 2B clinical trial studies showed that the GLP-1 agonist liraglutide reduced brain atrophy in regions involved in memory and decision-making by nearly 50% compared to the placebo [130,131]. Moreover, the GLP-1 analogue liraglutide has shown promising effects in improving cognitive function in both animal models and patients with AD by suppressing neuroinflammation through reduced microglial activation [132].

Despite these encouraging preclinical findings and the earlier data from patients, clinical trials evaluating GLP-1 receptor agonists in AD patients remain limited. Notably, the recent systematic review concluded that GLP-1 agonists, while showing some positive metabolic and neuroprotective effects, did not reduce beta amyloid and tau biomarkers and did not significantly improve the patient’s cognitive state [52]. Further studies are needed to clarify this still attractive approach, especially among patients with a comorbidity of AD and diabetes.

### 4.4. Coordinating Gut Motility, Secretion, and Barrier Integrity with Immunotherapy

Within the integrative view of the multiple interconnected functions of the gastrointestinal system that we hypothesize are disrupted in AD, in future therapies, it is necessary to coordinate various processes, such as the integrity of the intestinal barrier, gut motility, and secretion of digestive enzymes, as well as the proper activity of local nerves.

In this regard, a new promising role of immunotherapy in improving gut functions emerged recently, based on the CD4 therapy. Thus, Gómez de Las Heras et al. (2025) found, in the early senescing mouse model with T-cell failure associated with dysbiosis and a disrupted gut barrier integrity, that CD4 T cells or Treg-cell-enriched therapy prevented senescence features by restoring the gut barrier integrity [133]. Consistent with these results, Park et al. showed, in a mouse model, the beneficial results of autologous Treg cells in Parkinson’s disease [134].

This exciting new data suggests that similar T-cell-based immunotherapy could potentially also be used for patients with AD.

### 4.5. Targeting Descending Brain–Gut Mechanisms

Within the concept of the formation of *a vicious circle* in GBA, with disrupted descending neuronal signaling from the brain to the gut (Figure 3), the treatment options should be directed to improve such neuronal control. This should go along with therapeutics reducing amyloidosis and neuroinflammation in the brain, which can also, indirectly, optimize the complex gut activity through an improved descending control of the gut by the brain.

Consistent with this view, the direct activation of brain-to-gut communications could be based on vagus nerve stimulation. This technique is widely used already in various pathologies, including AD, where it shows a promising therapeutic effect [53,54,135,136]. Alternative to this could be the stimulation of the somatic–vagal–gastric reflex by electropuncture [137]. Notably, apart from the descending effect, vagus nerve stimulation can provide a beneficial ascending effect on brain functions, in particular, by activating the hypothalamic–pituitary–adrenal axis, which, as mentioned above, plays an important role in the control of the functional state of the gastrointestinal system.

Focused brain stimulation represents a range of dynamically developing techniques [138,139,140] also applicable to AD. For instance, in aged rats, the high-frequency deep stimulation of lateral hypothalamic areas reduced memory decline [141].

In general, non-invasive techniques are emerging, such as a battery of instrumental methods which demonstrate high potential as efficient tools in AD, alone or in combination with pharmacological approaches.

## 5. Discussion

In this narrative review, we present and discuss the multiple gut–brain communication pathways, known as the GBA, that support overall health but can become pathogenic in AD. This broad view is consistent with the emerging hypotheses that AD is not only a brain disorder but also a ‘systemic disease’ or organism-wide disorder [64].

The current understanding of GBA function is very broad, and in this review, we present many, but not all, potential GBA messengers, the number of which is constantly growing, updating our understanding of gut and brain function in health and disease. The current views on the role of the GBA in AD is largely based on preclinical studies, and the link between dysbiosis, gut signaling, and AD remains predominantly associative. This indicates the need for further research to clarify the molecular targets of gut messengers in the brain and their precise role in the pathogenesis of AD, taking into account the microbiota profile, combination with other treatments, and disease stage. We believe that, in addition to the accumulation of new data and obtaining better evidence, the development of theoretical approaches is also needed to address this complex topic.

Thus, we propose here that, in AD, the GBA is transformed from the *beneficial virtuous circle*, based on the positive loop, to the *harmful vicious circle* properties (Figure 3). The latter promotes the progression of AD and may be one of the factors causing the resistance of AD to the currently used treatment options [66]. Interestingly, disturbances in the descending neuronal/cognitive mechanisms in AD may also contribute to the formation of a vicious cycle.

This concept suggests, in general terms, that the treatment of AD requires breaking the harmful cycle and returning to the *virtuous circle* state. Practical steps that follow from this concept may involve minimizing the harmful ascending communication channels presented in this review and strengthening the weakened descending connections between the gut and the brain (Figure 3). When these processes are optimized and balanced, they can play a critical role in ensuring the normal digestion and absorption of not only essential nutrients, but also potentially anti-inflammatory substances that can be delivered to the brain.

For the purposes of personalized therapy, it would be ideal to first determine which of the multiple steps within the GBA are disrupted, to optimize personalized disease therapy according to the prevalence of these biomarkers. The latter suggests that the list of currently emerging biomarkers like plasma Aβ and p-tau217 [65] could be extended to gut-derived messengers, including SCFAs and BAs, microRNAs, EVs, and bacterial toxins.

In summary, the main translational impact of the proposed views suggests strategies for more advanced diagnostic tools and more complex treatments for AD and other neurodegenerative diseases, rather than the pure brain-targeted interventions used nowadays.

## 6. Perspective and Future Directions

- Updating our understanding of the pathogenesis of AD, in particular, with a more critical view on the Aβ hypothesis, further expanding the data on the risk factors for AD, and identifying new factors promoting neuroinflammation, including the flexible role of the microglia, should expand our knowledge on the complex interactions between the brain and gut in this disease.
- In this context, the promotion of the neuroprotective functions of microglia by intestinal metabolites is one of the promising perspectives for the treatment of AD.
- The future studies are expected to further update our understanding of multiple mechanisms on how the gut participates in the control of brain functions, and how this signaling is changed in AD. The novel molecular, subcellular, and cellular messengers, such as microRNAs, GLP-1/insulin, EVs, bacteria themselves and bacterial toxins, flagellin, and D-glucose signaling, require further exploration to be considered important players in the GBA in health and disease.
- It should be considered that the GBA is flexible, and many signaling pathways, like SCFAs and, especially, BAs, could be switched from beneficial to pathological, requiring a more in-depth assessment of the functional status of both the gut and brain.
- These current updates to the concept of the GBA offer better prospects for new treatments for AD, which should be considered as a disease of the whole organism, including the gastrointestinal system, among the other systems and tissues. In terms of the current review, the general goal could be to break the vicious cycle and return it to a beneficial virtuous circle.
- These options could be supplemented by the development of new technological approaches, such as synthetic bacteria, the in vivo editing of the microbiome, and AI tools.

## 7. Summary

In summary, we present a concept of the transformation of the beneficial virtuous circle into the harmful vicious circle, when an abnormal gut-to-brain branch vs. a weakened brain-to-gut branch of the GBA is considered as an essential factor, contributing to the development of the complex pathology of AD. We propose that the imbalance between these two branches of the GBA in patients with AD is due to converging factors such as an increased influx of pro-inflammatory factors from the gut into the brain through a permeable gut barrier and BBB, as well as abnormal neural and hormonal signaling. In return, the control of intestinal functions by the brain, ranging from abnormal eating behavior to descending channels of neural communication, further exacerbates this imbalance, contributing to, in addition to dysbiosis, the disruption of intestinal secretion, motility, and digestion. Although new GBA signaling pathways, such as those presented in this review, are continuously emerging, this concept may provide a theoretical basis for optimizing AD therapy by combining treatments targeting the gut and brain, rather than just the brain.

## Figures and Tables

**Table 1:** Beneficial and pathogenic contributors to the gut–brain axis and their role in Alzheimer’s Disease.

Beneficial Contributors				
Contributors	Primary Source	Main Effects on Gut–Brain Axis	Relevance to AD	References
Short-chain fatty acids (SCFAs: acetate, propionate, butyrate)	Fermentation of dietary fiber by anaerobes	Anti-inflammatory activity; strengthening BBB; support of microglial maturation and GI motility	Reduced SCFAs levels observed in AD; protective against neuroinflammation	[10,12,47]
Primary bile acids	Made in the liver from cholesterol	Modulates microbiota; signals via FXR/TGR5; supports gut barrier; reduces inflammation	Often reduced in AD; linked to dysbiosis, inflammation, and altered bile acid signaling	[48,49,50]
Serotonin (5-HT)	Enterochromaffin cells	Regulated motility, vagal signaling; local modulation of gut function and vascular control	Decreased gut production that affects motility and vagal signaling	[27,28,32]
GLP-1	L-cells	Enhancement of insulin signaling anti-inflammatory effects; neuroprotection	GLP-1 analogs show cognitive benefits and reduced AD pathology	[44,51,52]
Vagus nerve stimulation	Microbiotas metabolites, peptides	Activation of anti-inflammatory reflex; direct neural gut–brain communication	Potential to improve cognition and reduce inflammation; vagal activity reduced in AD	[53,54]
Normal gut motility	Enteric nervous system (ENS)	Prevents bacterial overgrowth; maintains homeostasis	Age-related motility decline contributes to AD-associated dysbiosis	[55,56]
**Pathogenic Contributors**				
**Contributors**	**Primary Source**	**Main Effects on Gut–Brain Axis**	**Relevance to AD**	**References**
LPS	Gram-negative bacteria	Strong pro-inflammatory action; increased BBB permeability; microglial activation	Detected in AD brain tissue; accelerates amyloid aggregation	[57,58,59]
Th 17 cells	Peripheral immune cells	Promotion of neuroinflammation; disruption of gut barrier integrity; altered microglial activity	Increased release IL-17 in the brain that leads to acceleration of AD progression	[15,16]
Bacterial extracellular vesicles (EVs)	Pathogenic gut bacteria	Transport inflammatory molecules, amyloid-like peptides; cross the BBB	May transport bacterial amyloids and promote inflammation in AD	[60,61]
Toxic bile acids (DCA, LCA)	Microbial metabolism	Induces oxidative stress and mitochondrial dysfunction	Associated with cognitive decline	[48,50,62]
Hypothalamic–pituitary–adrenal (HPA) overactivation	Stress-driven neuroendocrine signaling	Increased gut permeability, immune suppression, microbiota disruption	Chronic cortisol linked to memory loss and AD progression	[33,37,63]
Vagal nerve dysfunction	Aging, chronic inflammation, dysbiosis	Loss of anti-inflammatory reflex, impaired signaling	Associated with faster cognitive decline	[53,64]

## Data Availability

No new data were created or analyzed in this study. Data sharing is not applicable.
